# Successful transplantation of spermatogonial stem cells into the seminiferous tubules of busulfan-treated mice

**DOI:** 10.1186/s12978-021-01242-4

**Published:** 2021-09-23

**Authors:** Hossein Azizi, Amirreza Niazi Tabar, Thomas Skutella

**Affiliations:** 1 Faculty of Biotechnology, Amol University of Special Modern Technologies, P.O. Box 46168-49767, Amol, Iran; 2 Institute for Anatomy and Cell Biology, Medical Faculty, University of Heidelberg, Im Neuenheimer Feld 307, 69120 Heidelberg, Germany

**Keywords:** Germ cells, Spermatogonia, Stem cell transplantation, Busulfan

## Abstract

**Background:**

Spermatogonial stem cells (SSCs) in the testis are crucial for transferring genetic information to the next generation. Successful transplantation of SSCs to infertile men is an advanced therapeutic application in reproductive biology research.

**Methods:**

In this experimental research, both in vitro and in vivo characterization of undifferentiated and differentiated SSCs were performed by morphology—immunocytochemistry (ICC), immunohistochemistry (IMH), Fluidigm Real-Time polymerase chain reaction (RT-PCR) and flow cytometry analysis. The isolated SSCs were finally microinjected into the rete testis of busulfan-treated mice.

The compact undifferentiated and more loosely connected round differentiated SSCs were isolated during testicular cell expansion from their specific feeder layer.

**Results:**

ICC analysis indicated high and low expression levels of Zbtb16 in undifferentiated and differentiated germ cells. Also, IMH analysis showed different expression levels of Zbtb16 in the two different germ stem cell populations of the testicular tissue. While Fluidigm RT-PCR analysis indicated overexpression of the TAF4B germ cell gene, the expression of DAZL, VASA, and Zbtb16 were down-regulated during the differentiation of SSCs (*P* < 0.05). Also, flow cytometry analysis confirmed the significant downregulation of Itgb1 and Itga4 during differentiation. By transplantation of SSCs into busulfan-treated NOD/SCID mice, GFP-labeled sperm cells developed.

**Conclusions:**

In the current study, we performed a transplantation technique that could be useful for the future microinjection of SSCs during infertility treatment and for studying in vivo differentiation of SSCs into sperm.

## Plain language summary

Spermatogonia (SSCs) in the testis transmit genetic information to the next generation. Successful SSC transplantation into infertile men is an advanced therapeutic application in reproductive biology research. In this experimental research, both in vitro and in vivo characterization of undifferentiated and differentiated SSCs were performed by morphology—immunocytochemistry (ICC), immunohistochemistry (IMH), Fluidigm Real-Time polymerase chain reaction (RT-PCR) and flow cytometry analysis. The isolated SSCs were finally microinjected into the rete testis of busulfan-treated mice. ICC analysis indicated high and low expression levels of Zbtb16 in undifferentiated and differentiated germ cells. IMH analysis showed different expression levels of Zbtb16 in both populations. Fluidigm RT-PCR analysis indicated overexpression of the TAF4B germ cell gene and the down-regulated expression of DAZL, VASA, and Zbtb16 during SSCs differentiation of (P < 0.05). Flow cytometry analysis confirmed the significant downregulation of Itgb1 and Itga4 during differentiation. By transplantation of SSCs into busulfan-treated NOD/SCID mice, GFP-labeled sperm cells developed. We performed a transplantation technique that could be useful for the future microinjection of SSCs during infertility treatment and for studying in vivo differentiation of SSCs into sperm. Data analysis confirmed that zbtb16 is expressed in the undifferentiated germ cells located on the basal membrane of seminiferous tubules and SSCs in vitro. Also, spermatogenesis was resumed, and fertility improved after transplantation of undifferentiated cells into busulfan-treated mice; thus, improvements in vitro SSCs transplantation, isolation and culture would be helpful in future clinical treatments to solve the reproductive problems of families influenced by infertility.

## Background

Spermatogenesis is an organized complex process within the seminiferous tubules, which is started by undifferentiated spermatogonia located on the basement membrane of the seminiferous tubule [1]. During this process, regulated proliferation and differentiation of undifferentiated spermatogonia determine the long-term production of mature spermatozoa for the transmission of genetic information to the subsequent generation [2]. In accordance, undifferentiated spermatogonia continuously undergo self-renewal for the maintenance of the progenitor state [3]. In vivo conditions for normal spermatogenesis process require regulatory mechanics mainly controlled by endocrine signals from testicular somatic cells such as peritubular myoid cells (PTCs), Leydig cells, and Sertoli cells (SCs) [4].

PTCs are arranged in a discontinuous cell layer and have characteristic features such as large and flat shape. PTCs produce interleukins (ILs), Insulin-like growth factor 1 (IGF-1), basic fibroblast growth factor (bFGF), and PModS. PModS (peritubular modifies Sertoli) is a modulator molecule for androgen-binding protein, inhibin, and transferrin by SCs [5–7].

Leydig cells are located between blood vessels and seminiferous tubules and express the luteinizing hormone (LH) receptor. After binding to its ligand, Leydig cells produce testosterone via a biochemical complex process triggered with cholesterol. Then, the release of testosterone into seminiferous tubules provides a particular environmental condition for normal spermatogenesis [8, 9].

One of the most important somatic cells located in the epithelium of seminiferous tubules are SCs, which have a strategic position necessary for their functions [10]. SCs are responsible for the creation of a physiologic barrier named blood testis barrier (BTB). BTB has a selective penetration to provide a specific environment for spermatogenesis needs [11]. The physical role of the BTB is to maintain the concentration of ions and molecules needed to be present in the seminiferous tubules [12]. In addition, BTB plays an immune role to protect new spermatid and sperms produced in seminiferous tubules from self-immune system [13]. Also, SCs are modulated by hormonal systems such as response to follicle-stimulating hormone [14].

Only a few factors are known to provide the regulation of SSCs functions [15]. Several cell surface markers, such as octamer-binding transfer Factor-4 (Oct-4), glial cell line derived neurotrophic factor family receptor alpha 1 (GFRa1), SALL4, LIN28, and c-Kit were used to understand more characteristic features of undifferentiated and differentiated spermatogonia by fluorescence‐activated cell sorting (FACS) and immunohistochemistry assays [4]. According to Dann et al., Oct-4 is required for SSCs maintenance and consequently plays a critical role in the pluripotency of embryonic stem cells [15]. It has been reported that GFRa, SALL4 and LIN28 are expressed in undifferentiated spermatogonia whereas c-Kit is expressed in differentiated spermatogonia [16–18]. These results have increased the knowledge of the testicular SSC population and consequently helped to improve the therapeutic strategies against infertility. The transition from undifferentiated to differentiated spermatogonia is highly determined by gene expression [19]. Thus, characterization by different nuclear staining methods and expression of molecular markers are necessary to improve future reproductive biology developments.

In this investigation, specialized markers, including Zbtb16, TAF4B, DAZL, and VASA were used for immunohistochemistry/immunocytochemistry and real-time PCR analyses. Flow cytometry analysis was applied for Itgb1 and Itga4. Furthermore, isolated germ cells were injected into busulfan-treated NOD/SCID mice by transplantation as a functional biological assay.

## Methods

### Enzymatic digestion and culture of testicular cells

Testicular cells from C57BL/6 mouse strain were placed in an enzymatic digestion solution containing DNase (0.5 mg/ml) (Sigma Aldrich), collagenase (0.5 mg/ml) (Sigma Aldrich), and dispase (0.5 mg/ml) (Sigma Aldrich) in HBSS buffer (PAA, USA). Digested testicular cells were filtered through a cell strainer and cultured in the GSCs culture media containing the StemPro-34 medium, 1% L-glutamine (PAA, USA), 1% N2-supplement (Invitrogen, USA), 6 mg/ml of D + glucose (Sigma Aldrich, USA), 5 µg/ml of bovine serum albumin (BSA) (Sigma Aldrich, USA), 1% penicillin/streptomycin (PAA, USA), 30 ng/ml of estradiol (Sigma Aldrich, USA), 0,1% ß-mercaptoethanol (Invitrogen, USA), 1% non-essential amino acids (PAA, USA), 60 ng/ml of progesterone (Sigma Aldrich, USA), 10 ng/ml of FGF (Sigma Aldrich, USA), 1% MEM vitamins (PAA, USA), 100 U/ml of human LIF (Millipore), 20 ng/ml of epidermal growth factor (EGF), (Sigma Aldrich, USA), 8 ng/ml of GDNF (Sigma Aldrich, USA), 1% ES cell qualified FBS, 30 µg/ml of pyruvic acid (Sigma Aldrich, USA), 1 µl/ml of DL-lactic acid (Sigma Aldrich, USA), and 100 µg/ml of ascorbic acid (Sigma Aldrich, USA) at 37 °C and 5% CO2 in air.

### Immunohistochemistry

Testicular tissue was picked up after decapsulation of tunica albuginea, washed with PBS, and fixed in 4% paraformaldehyde. The tissue was dehydrated during tissue processing and surrounded in Paraplast Plus. In the next step, tissues were cut usually around 8–10 µm thickness with a microtome. Testicular tissue sections were mounted on Hydrophilic Plus slides and stored at room temperature until use. During immunohistofluorescence staining process, slides were washed by xylene and water was replaced slowly through a series of decreasing ethanol concentrations. Before staining, antigen retrieval was done by the heat-induced epitope retrieval (HIER) method at 95 °C for 20 min, and non-specific binding sites in the tissue sections were blocked with 10% serum/0.3% Triton in PBS. The characterization of immunofluorescence staining for these sections was followed as described above.

### Immunocytochemistry

Isolated testicular SSCs were fixed with 4% paraformaldehyde, permeabilized with 0.1% Triton X-100/PBS, blocked with 1% BSA/PBS, and then incubated with the primary antibody Zbtb16 (Calbiochem OP128). The process was followed with overnight incubation (usually ~ 16 h) of fluorochrome species-specific secondary antibody at 4 °C. The labeled cells were identified by simple nuclear counterstain with 0.2 µg/ml of 4', 6-diamidino-2-phenylindole (DAPI) dye. Labeled positive cells with antibodies were considered by a confocal laser scanning microscope (Zeiss LSM 700), and images of cells were obtained using a Zeiss LSM-TPMT camera.

### Flow cytometric analysis

After cell viability determined by trypan blue staining, cells were resuspended in the PBS/FBS staining buffer and incubated with cell surface primary antibodies Itga4 (R&D Systems, FAB13501A) and Itgb1 (MACS, 130-096-356), which was conjugated with fluorochrome (APC) magnetic-activated cell sorting (MACS) for 1 h. Samples were subjected to a wash procedure and flow cytometric analysis was performed with a BD-FACS Calibur Flow Cytometer. Acquired results were analyzed with the BD CellQuest Pro software.

### Fluidigm RT-PCR

The expression levels of the *DAZL* (Mm00515630_m1), *VASA* (Mm00802445_m1), *TAF4B* (Mm01254136_m1) and *Zbtb16* (Mm01176868_m1) genes in the SSCs and TSC cells were examined by the Fluidigm biomark system. SSCs and TSCs were picked up with a micromanipulator technique and lysed with a lysis buffer solution containing 9 μl of RT-PreAmp Master Mix (5.0 μl of Cells Direct 2 × Reaction Mix, Invitrogen, USA), 2.5 μl of 0.2 × assay pool, 1.3 μl of TE buffer, and 0.2 μl of RT/Taq Superscript III (Invitrogen, USA). The amounts of amplified product of RNA-targeted copies were then examined with TaqMan RT-PCR on the BioMark Fluidigm RT-PCR system. Samples were analyzed in two technical repeats. The Ct values were calculated using the Excel and GenEx software.

### Transplantation

Cells were transfected with the enhanced green fluorescent protein (EGFP) gene (COP-GFP). For site-specific transplantation into the tubuli seminiferi contorti of the testis, approximately 10 µl of the donor cell suspension (containing 0.5–1 × 105 SSCs) was injected into the rete testis of male SCID NO mice treated with busulfan (44 mg/kg) at 6 weeks of age [67]. Adult recipient mice were anesthetized with ketamin (100 mg/kg) – xylazine (20 mg/kg) solution. Testes were fixed in paraffin and cryo-blocks and sections were analyzed by confocal microscopy 4–8 week after transplantation. Sperm cells were kept at − 20 °C after isolation from the glandula epididymidis and then fixed on slides. Similarly, sperm cells from EGFP mouse samples were isolated from the glandula epididymidis to compare with our samples (These experiments were performed in Royan Institute, Tehran, Prof. Baharvand´s group). Mice were treated with busulfan at a dose of 30 mg/kg for 3 weeks to be rendered infertile. All mouse experiments were conducted according to the NWSUAF guideline of Animal Care and Use Committee.

### Statistical analysis

The expression of CD-117 in neonate GSCs, adult GSCs, and TSC groups was analyzed using one-way analysis of variance (ANOVA), followed by the Tukey's post-hoc test and compared with the non-parametric Mann–Whitney’s test. The difference between SSCs and TSCs was statistically reliable if P < 0.05.

### Ethical consideration

In the current investigation, animal experiments were approved (Ir.ausmt.rec.1398.03.07) by the Ethics Committee of Amol University of Special Modern Technologies.

## Results

**Fig. 1:**
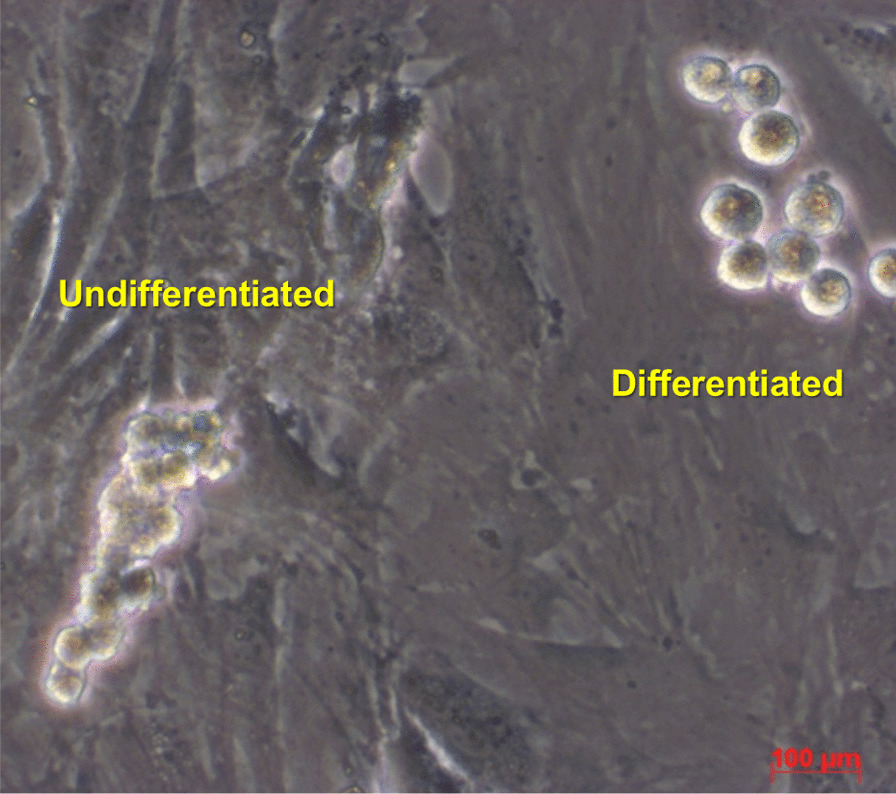
Expansion of different testicular cell types on the feeder layer. Bright field photo of the two cell type colonies. Two distinct types of differentiated and undifferentiated cells are obviously shown. Undifferentiated SSCs grow in more compact assemblies in comparison to more loosely connected differentiated SSCs

In this experimental study, two morphological distinguishable cell types, namely differentiated and undifferentiated SSC obviously expanded after enzymatic digestion on the feeder layer of the cell culture wells (Fig. 1).

**Fig. 2:**
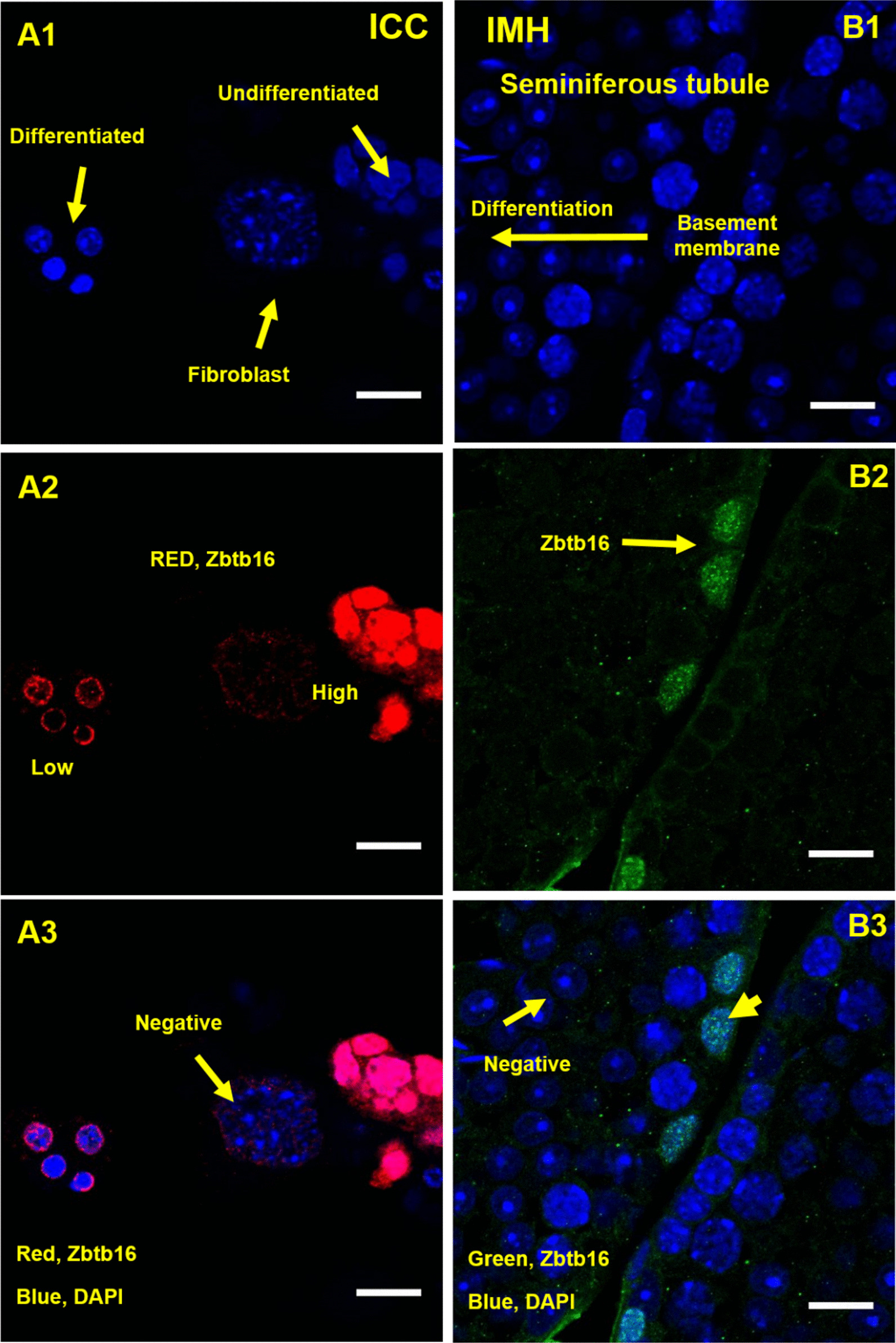
Immunohistochemistery (IMH) and immunocyto chemistery (ICC) analyses of zbtb16 indicates high expression of the zbtb16 in cells located on the basement membrane and also in the undifferentiated germ stem cells of the seminiferous tubules in culture (**A2**, **B2**). Merging blue DAPI and zbtb16 (**A3**, **B3**) (Scale bar = 50 μm)

The spermatogonial stem cells marker zbtb16 marker was localized in cells of the basal compartment of the seminiferous tubules of mice by immunohistochemical (IMH) and in cell culture by immunocytochemical (ICC) analyses. ICC analysis demonstrated that the Zbtb16 expression in cultured cells was higher in the undifferentiated than in differentiated cells (Fig. 2: A1, A2, A3). Also, IMH analysis indicated a high expression of the Zbtb16 in cells directly located on the basement membrane while a low expression was observed in the differentiated compartment (Fig. 2: B1–B3).

**Fig. 3:**
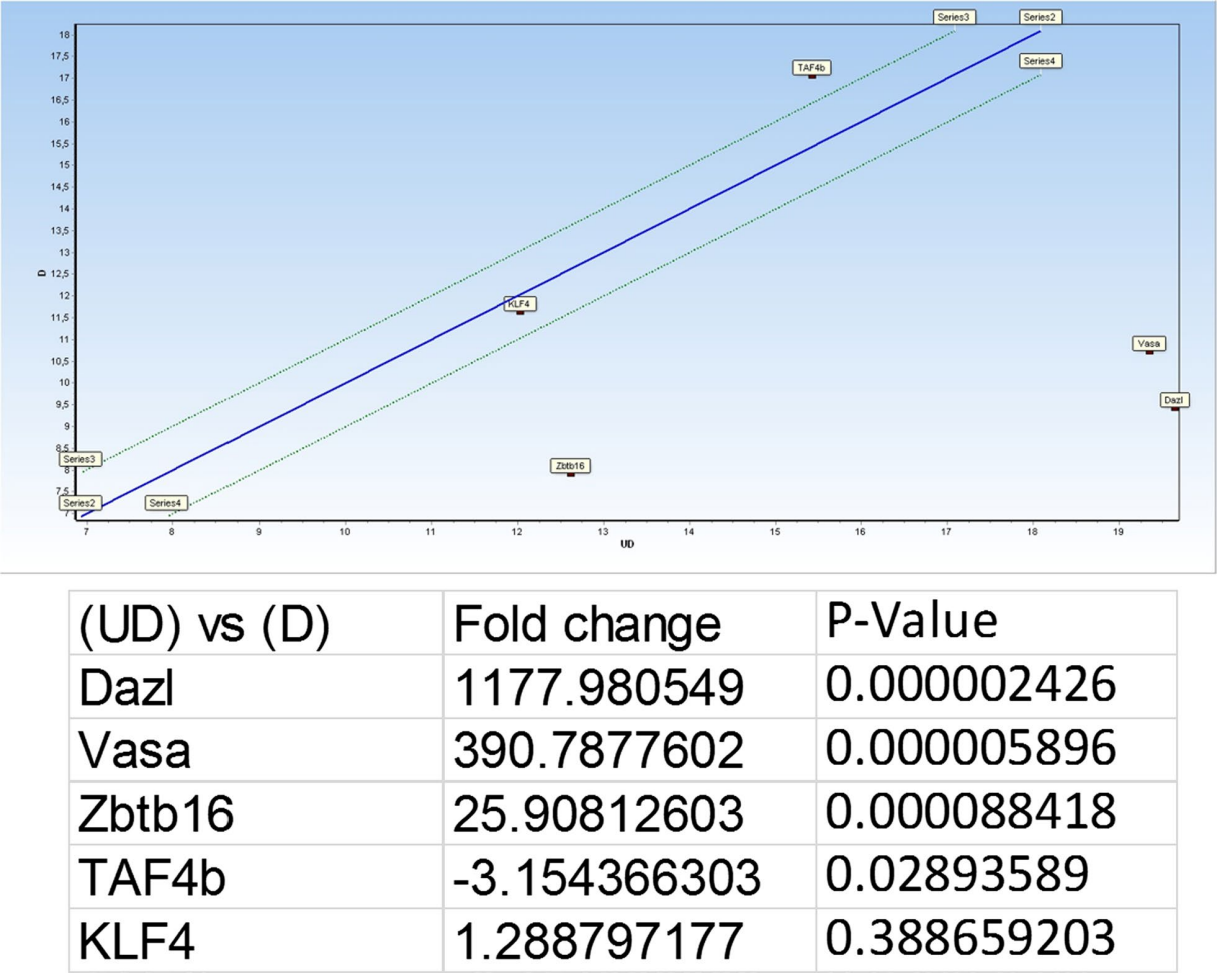
Fluidigm RT-PCR analysis indicates high expression of DAZL, VASA and Zbtb16 in undifferentiated (UD) spermatogonial cells, while higher expression of TAF4B was observed in differentiated (D) germ cells (P < 0.05)

**Fig. 4:**
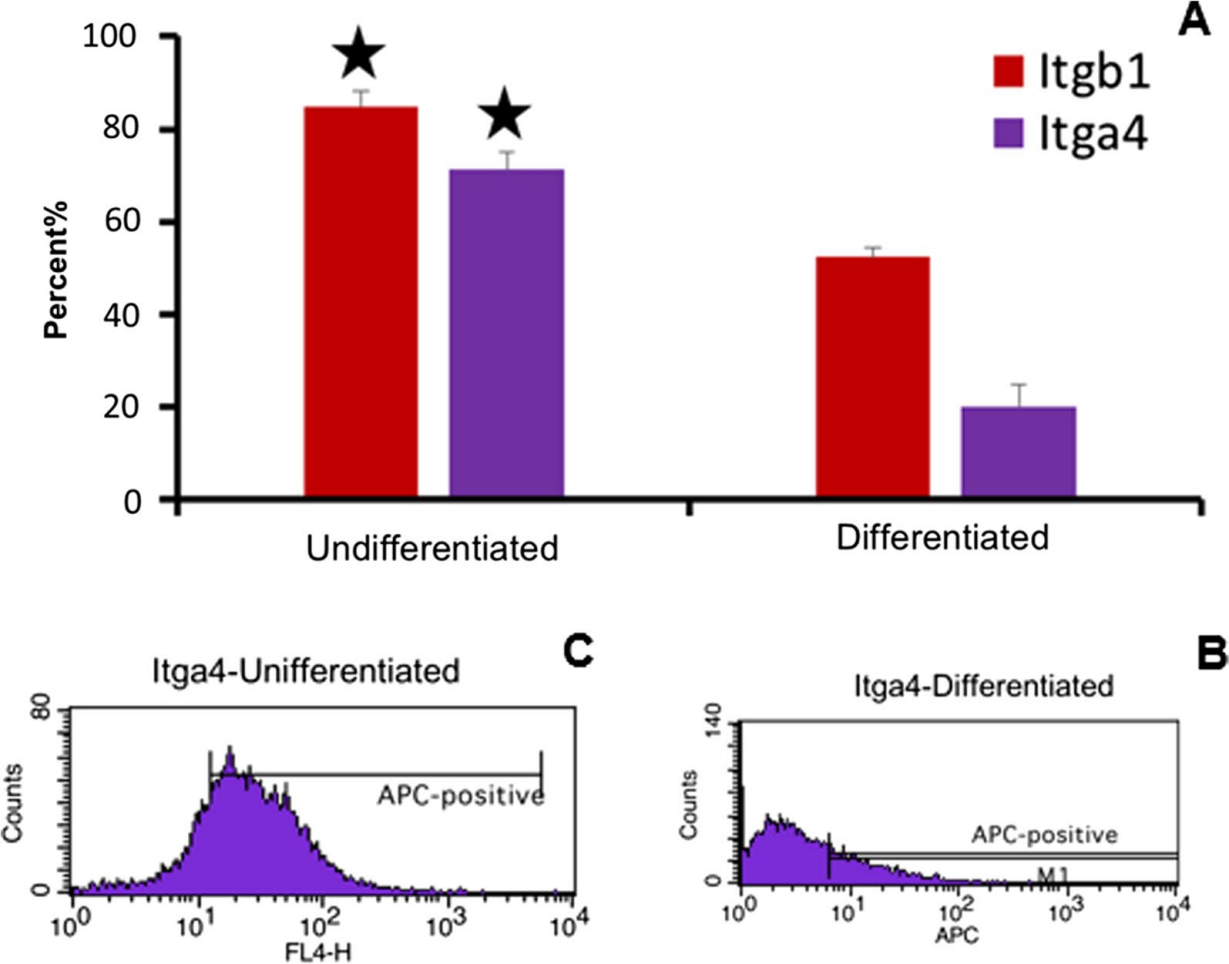
Flow cytometry analysis. Significant down-regulation of Itgb1 and Itga4 during differentiation of SSCs. Comparative analysis of Itgb1 an Itga4 showed a significant expression in undifferentiated relative to differentiated cells (**A**). Itga4 expression diagram in differentiated (**B**) and undifferentiated (**C**) cells

The results of Fluidigm RT-PCR analysis showed up-regulation of Zbtb16 and the other two SSC marker VASA and DAZAL in the undifferentiated SSCs, whereas a highler expression of TAF4B is shown in more differentiated SSC (*P* < 0.05) (Fig. 3). KLF4 showed no different beween the two cells types. Significant high regulation of Itgb1 and Itga4 was detected in undifferentiated cells indicated by flow cytometry analysis (Fig. 4).

**Fig. 5:**
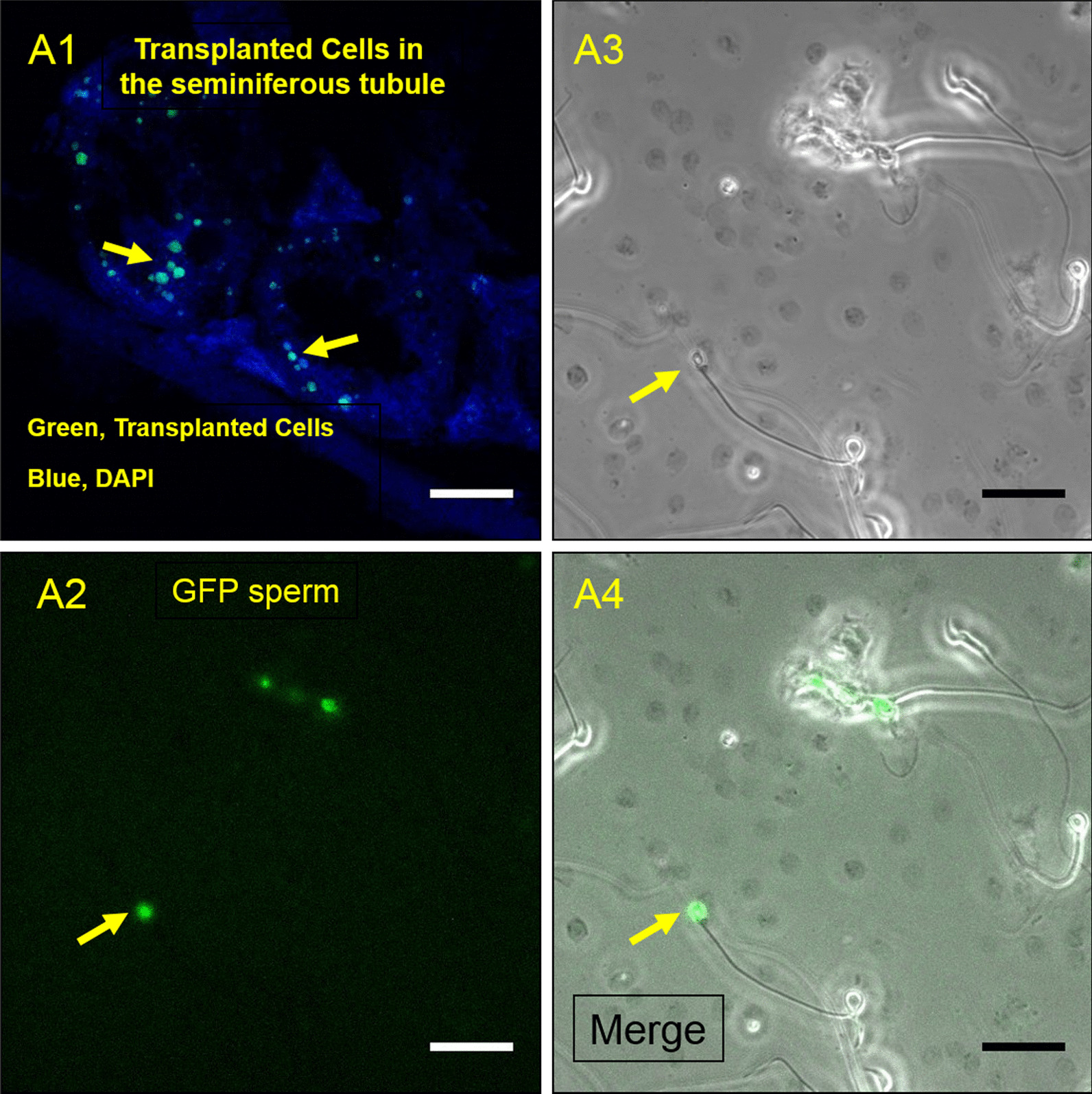
Transplantation of SSCs in vivo. The GFP (Green Fluorescent Protein) label proved that the injected cells differentiated into sperm. Localisation of the labeled injected SSCs in the basal compartment of the seminiferous tubules (**A1**). Bright field analysis (**A3**, **A4**) and merging with GFP (**A2**) showed that injected cells developed to sperm

Spermatogenesis in the busulfan-treated mice improved significantly after transplantation of isolated SSCs (*P* < 0.001) (Fig. 5). The green fluorescent protein (GFP) labeling of injected cells verified their differentiation into sperm (Fig. 5: A2, A4).

## Discussion

The application use of a multivariable analysis, including DAZL, VASA, Zbtb16,TAF4b, Itgb1 and Itga4 indicated a significant differential expression of all these markers except KLF4.

In this present examination, zbtb16 was used as the primary marker to discriminate the differentiated from undifferentiated SSCs. The Zbtb16 or PLZF (promyelocytic leukaemia zinc finger) is a transcriptional factor and intracellular marker which contained nine Kruppel-type zinc finger domains at the carboxyl terminus that binds to the zinc finger domain of DNA and is involved in cell cycle progression.. [20, 21]. Also, Buaas et al. have shown that targeted disruption of the zbtb16 caused male sterility, so it is considered an essential factor in the development and spermatogenesis in mammalian testis [22].

According to the investigation by Haixin Li et al., one of the essential factors for translation regulation in vertebrate spermatogenesis is Deleted in azoospermia-like (DAZL) [23].

Deleted azoospermia (DAZ) is one of the RNA-binding proteins of germ cells that includes DAZ, deleted azoospermia-like (DAZL), and BOULE. Only DAZL is expressed in both genders, but both the DAZ and BOULE are required for fertility in males [23–25].

DEAD-box helicase (DDX4 or vasa) is also an essential factor in maintaining germ cells. Most recently, vasa expression has been reported in spermatocytes and round spermatids by IMH analysis [26].

In addition, it has recently been reported that disruption of TAF4b and ZFP628 by crisper-cas9 induces infertility in male mice [27]. In addition to the above, Yamanaka and et al. has been reported that in mammals, a zinc finger transcription factor, known as kruppel-like factor 4 (KLF4), plays an essential role in reprogramming somatic cells to induced pluripotent stem cells. Furthermore, KLF4 has been reported to regulate early spermatogenesis and maintain the functions of the testis [28, 29].

In the recent decade, several investigations on animal models (such as mice, monkeys, bovine, and alpaca) have sought to introduce proper markers for identification of SSCs as the main cells in male reproductive life. A recent study has suggested that GFRa can be used as the bovine undifferentiated germ cell marker [30]. Also in another recent study, the zbtb16 has been used as the molecular marker of SSCs in alpaca [31]. Accordingly, the ICC analyses of isolated spermatogonial cells were used here, after in vitro plating, to prove that the zbtb16 is a specific marker to SSCs. As expected, our final results demonstrated that the zbtb16 is highly expressed in the differentiated cells among other cells in the seminiferous tubules. Also, the cells with the low expression level of zbtb16 (undifferentiated) were transplanted into busulfan treated mice. Consequently, the injected cells underwent spermatogenesis and differentiated into sperm.

Similarly, Kadam et al. have shown a significantly better tubular fertility index in all transplanted models three months after transplantation of SSCs into busulfan mice [32]. Successful transplantation of germ cells will hopefully open the way for future clinical treatments against infertility [33, 34]. Because infertility is a widespread global issue, knowledge of germ cell differentiation is beneficial for understanding the functional and regulatory mechanisms of spermatogenesis and reproductive strategies.

## Conclusion

Our data analysis confirmed that zbtb16 is expressed in the undifferentiated germ cells located on the basal membrane of seminiferous tubules of testes and in stem cell SSCs in vitro. Beside this observation, spermatogenesis was resumed, and fertility improved after transplantation of undifferentiated cells into busulfan-treated mice. Therefore, improvements in vitro isolation and culture of SSCs and their transplantation into infertile models would be helpful in future clinical treatments to solve the reproductive problems of families influenced by infertility.

## Data Availability

The data sets analyzed for the current study are available from the corresponding author on reasonable request.

## Abbreviations

SSC: Spermatogonial Stem cell
TSC: Testicular stromal cell;
PTC: Peritubular myoid cells
SCs: Sertoli cells
ILs: Interleukins
IGF-1: Insulin-like growth factor 1
bFGF: Basic fibroblast growth factor
PModS: Peritubular modifies Sertoli
LH: Luteinizing hormone
BTB: Blood testis barrier
Oct-4: Octamer-binding transfer Factor-4
GFRa1: Glial cell line derived neurotrophic factor family receptor alpha 1
FACS: Fluorescence‐activated cell sorting
IMH: Immunohistochemical
ICC: Immunocytochemical
GFP: Green fluorescent protein
